# Supplementary material for: The interactome of intact mitochondria by cross-linking mass spectrometry provides evidence for coexisting respiratory supercomplexes
Source: Mol Cell Proteomics. 2017 Dec 8;17(2):216–32. doi: 10.1074/mcp.RA117.000470 (PMC5795388; doi:10.1074/mcp.RA117.000470)
Supplement: Supplemental Data [file supp_RA117.000470_133922_0_supp_23978_fzffwf.zip › spectra_annotation/mito_DR_spectra_annotation/264-1-7-1-3-1.pdf]

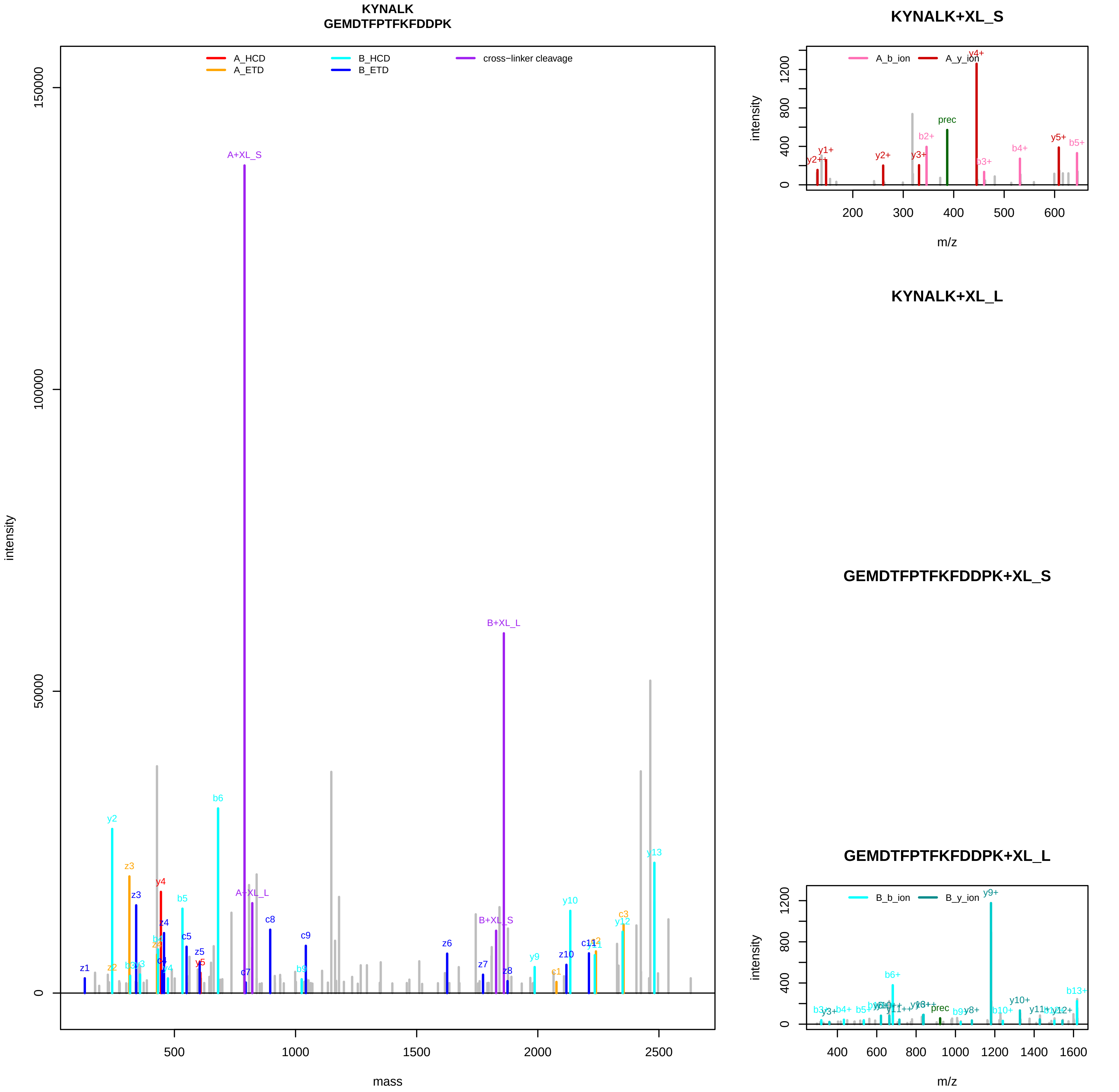

KYNALK
GEMDTFPTFKFDDPK
A_HCD
A_ETD
B_HCD
B_ETD
cross−linker cleavage
intensity
mass
150000
100000
50000
0
500
1000
1500
2000
2500
A+XL_S
A+XL_L
B+XL_L
B+XL_S
y2
z3
y4
z2
z1
z4
b5
c5
z5
y5
b6
c7
c8
b9
c9
z6
z7
z8
y9
z10
y10
c11
y11
y12
c3
y13
c1
KYNALK+XL_S
A_b_ion
A_y_ion
prec
y1+
y2+
y3+
y4+
y5+
b2+
b3+
b4+
b5+
m/z
200
300
400
500
600
1200
800
KYNALK+XL_L
GEMDTFPTFKFDDPK+XL_S
GEMDTFPTFKFDDPK+XL_L
B_b_ion
B_y_ion
y9+
b6+
b13+
y10+
b10+
y8+
b9+
b3+
y3+
b4+
b5+
1000
1400
1600
